# Ecology's Big, Hot Idea

**DOI:** 10.1371/journal.pbio.0020440

**Published:** 2004-12-14

**Authors:** John Whitfield

## Abstract

Can the emerging field of metabolic ecology explain all life's patterns in one unifying theory, from the metabolic rate of a shark to why biodiversity peaks at the equator?

Life is complicated. It comes in all sorts of shapes, sizes, places, and combinations, and has evolved a dizzying variety of solutions to the problem of carrying on living. Yet look inside a cell and life takes on, if not simplicity, then at least a certain uniformity—a genetic system based around nucleic acids, for example, and a common set of chemical reactions for turning food into fuel. And looked at in broad swathes, life shows striking generalities and patterns. Every mammal's heart will beat about one billion times in its lifetime. Both within and between species, the density of a population declines in a regular way as the size of individuals increases. And the number of species in all environments declines as you move from the equator towards the poles.

Wouldn't it be good if there were a simple theory that used life's shared fundamentals to explain its large-scale regularities, via its diversity of individuals? In the past few years, a team of ecologists and physicists have come up with just such a theory. At its heart is metabolism: the way life uses energy is, they claim, a unifying principle for ecology in the same way that genetics underpins evolutionary biology. They believe that energy use, in the form of metabolic rate, can be understood from the first principles of physics, and that metabolic rate can explain growth, development, population dynamics, molecular evolution, the flux of chemicals through the environment, and patterns of species diversity—to name a few.

The work, its originators insist, is not a theory of everything for biology, or even ecology. But it can often seem that way. “We're making advances on a broad range of questions almost on a weekly basis,” says James Gillooly, of the University of New Mexico, Albuquerque. “We've been having an awful lot of fun.”

## Beneath the Surface

Metabolic ecology, as it has become known, is still controversial. Some think its mathematical foundations are unsound, and that it explains nonexistent trends. It also divides researchers on philosophical lines—those that see life's patterns as fundamental versus those who think that variation is the key, those who think that simple, general ideas can help us understand nature versus those who think that complicated problems require complicated answers. A lot is riding on the debate: “If the theory is right, it's one of the most significant in biology for a long time,” says ecologist David Robinson of the University of Aberdeen. “It would provide a common functional basis for all biodiversity.”

Scientists have known for nearly two centuries that larger animals have relatively slower metabolisms than small ones. A mouse must eat about half its body weight every day not to starve; a human gets by on only 2%. The first theories to explain this trend, developed in the late nineteenth century by the German nutritionist Max Rubner and the French physiologist Charles Richet, were based on the ratio between an animal's surface area, which changes with the square of its length, and its volume, which is proportional to its length cubed. So large animals have proportionately less surface area, lose heat more slowly, and, pound for pound, need less food. The square-versus-cube relationship makes the area of a solid proportional to the two-third power of its mass, so metabolic rate should also be proportional to mass^2/3^. For many years, most biologists thought that it was.

But in 1932, Max Kleiber, an animal physiologist working at the University of California's agricultural station in Davis, re-examined the question, and found that, for mammals and birds, metabolic rate was mass^0.73^—closer to three quarters than two thirds. Kleiber looked at animals ranging in size from a rat to a steer. By the mid-1930s, other workers had put together a “mouse to elephant” curve that supported the three-quarter-power law, and by the 1960s, the plot had been extended for everything from microbes to whales, still seeming to show the same relationship. Quarter-power scaling also began to stretch beyond metabolic rate. Biological times, such as lifespan and heart rate, were found to be proportional to mass^1/4^, and fractions related to one-quarter show up in other scaling relationships: the diameter of the aorta and tree trunks is proportional to mass^3/8^, for example.

It was, however, much harder to find a theoretical reason for why metabolic rate should be proportional to mass^3/4^—and more generally, why quarter-power scaling laws should be so prevalent in biology. The impasse meant that by the mid-1980s interest in scaling had waned. But it sparked back into life in 1997, when two ecologists—James Brown of the University of New Mexico, Albuquerque, and his graduate student Brian Enquist, now at the University of Arizona, Tucson—and a physicist, Geoffrey West of the Santa Fe Institute, developed a new explanation of why metabolic rate should equal the three-quarter power of body mass.

West, Brown, and Enquist's theory is based on the structure of biological distribution networks, such as blood vessels in vertebrates and xylem in plants. The trio assumed that metabolic rate equals the rate at which these networks deliver resources, and that evolution has minimized the time and energy needed to get materials from where they are taken up—the lungs or roots, for example—to the cells. They also assumed that, although organisms vary greatly in size, the terminal units in their distribution networks, such as blood capillaries or leaf stalks, do not.

Bigger plants and animals take longer to transport materials, and so use them more slowly. In West, Brown, and Enquist's model, the maximally efficient network that serves every part of a body has a fractal structure, showing the same geometry at different scales. And the number of uniform terminal units in such a network—and so the rate at which resources are delivered to the cells—is proportional to the three-quarter power of body mass.

## Pattern versus Variation

Whether metabolic rate really varies with the three-quarter power of body mass is still debated—some researchers still favor two-thirds, others think that no one exponent fits all the data—but a majority of biologists favor three-quarters. And whether the fractal theory really explains the relationship of metabolic rate to body size is also still contentious. In the most wide-ranging critique so far, published this April, two Polish researchers, Jan Kozlowski, of Jagiellonian University, Krakow, and Marek Konarzewski of the University of Bialystok, claimed that the theory's maths could not simultaneously contain both uniform terminal units and three-quarter-power scaling, that large animals built along such lines would have more blood than their bodies could contain, that biological scaling laws were not built around quarter powers, and that biological networks were not generally branching fractals.

“I don't believe there's anything to explain—there's no universal scaling exponent,” says Kozlowski. He is also struck by what is left unexplained when size is accounted for: animals of the same size can still show more than an order of magnitude variation in metabolic rate. “What's striking in nature is the variability,” he says. “There are regularities that call for explanation, but that doesn't mean ignoring the variability is correct.” Kozlowski is the co-author of a theory that relates metabolic rate to cell size and the amount of DNA an organism has, one of several alternative explanations of the scaling of metabolic rate published since West, Brown, and Enquist's model.

The criticisms are serious, says Robinson. “The jury is out—questions about the fundamental maths are worrying a few people.” On the other hand, he says, West, Brown, and Enquist's model seems a plausible template for designing an organism, and its predictions fit real-world data remarkably well. Whether this fit truly captures the physical and chemical mechanisms underlying the patterns remains to be seen; Robinson hopes that criticism can strengthen West, Brown, and Enquist's model, perhaps leading to a new, improved theory.

The metabolic theory's authors are not budging. “We've yet to see a criticism we feel we can't answer pretty readily,” says Brown. Kozlowski and Konarzewski's arguments are based on a misreading of the work, he says, and criticisms that focus on one aspect, such as the structure of mammalian vascular systems, miss the key point, which is generality: “If we're wrong on quarter powers, why do they keep showing up in everything from life-history processes to evolutionary rates?”

## From Sharks to Tomatoes

After accounting for size, Brown's group turned its attention to the second most important influence on metabolism: temperature. The effect is exponential, and a 5 °C rise in body temperature equals a roughly 150% rise in metabolic rate. The team built an equation for metabolic rate that combined the mass^3/4^ term with the Boltzmann factor. The latter is an expression of the probability that two molecules bumping into each other will spark a chemical reaction. The higher the temperature, the greater the probability, and the faster the reaction.

**Figure 1:**
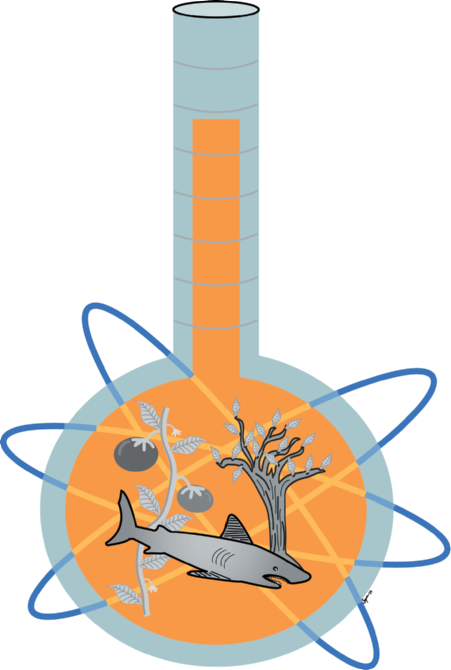
After Correcting for Body Size and Temperature, the Metabolic Rates of a Shark, a Tomato Plant, and a Tree Are Remarkably Similar (Illustration: Sapna Khandwala)

Adding temperature explained much of the variation in metabolic rate that remained after adjusting for size. It also explained some of the metabolic differences between groups. For example, a reptile has a slower metabolic rate than a mammal of the same size. But adjusting for its lower body temperature removes much of the difference, suggesting that the two groups share fundamental metabolic processes. The same even goes for plants and animals. “When you correct for size and temperature, the metabolic rates of a shark, a tomato plant and a tree are remarkably similar,” (Figure 1) says Gillooly, who joined Brown's group as a grad student to work on the temperature question. It's not yet clear what the activation energy represents, says Gillooly. It could be a kind of average for all the hundreds of chemical reactions in metabolism, or maybe the energy needed to get over one crucial hump in the path.

The metabolic theory's third component, resources, is also something of a black box at this stage. Nutrient supply, the team reasons, is the next most important determinant of metabolic rate, and will account for some of the remaining unexplained variation. As with temperature, the overall effect could be a balance of many processes, or it could be due to one limiting element—the growth of lake phytoplankton is often limited by phosphorus, for example, while for marine phytoplankton iron is usually the crucial nutrient. “It's a work in progress,” says Brown. “But our vision for a metabolic theory of life is ultimately going to include material resource limitation.”

**Figure 2:**
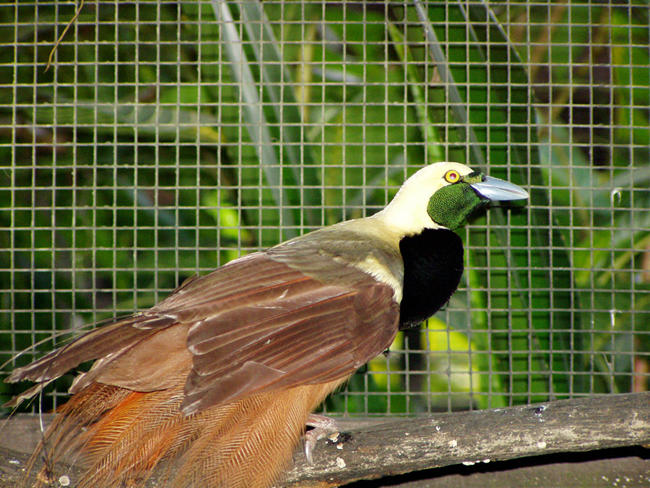
Adult Male Raggiana Bird of Paradise (Paradisaea raggiana) Body size, altitude, and diet account for 99.0% of the variation in the metabolic rates of birds of paradise. (Photo: Brian McNab)

These three things still do not account for all the variation in metabolic rate, but more detailed knowledge of species can yield more precise predictions. Using body size, altitude, and diet, Brian McNab, of the University of Florida, Gainesville, has explained 99.0% of the variation in metabolic rate for birds of paradise (Figure 2), and 99.4% of the rate variation in leaf-nosed bats. Nevertheless, when McNab sees attempts to explain variation in metabolism using a few parameters applied across a wide range of sizes and taxonomic groups, what isn't explained strikes him as forcefully as what is.

“I have serious reservations as to whether there is a single relationship for body size and metabolic rate,” he says. “I think we will be able to find generalizations in ecology, but they're not going to be simple—there will be a bunch of clauses and restrictions, and animals have a lot of ways to bend the rules.”

No theory matches data exactly, Brown points out; having a baseline prediction for metabolism lets you identify exceptional cases worthy of further investigation. Viewed from this angle, the metabolic theory is a kind of null hypothesis of how organisms work. “Until you have a theory that makes a prediction, you don't know how to interpret any of the variation,” says Brown. And, he adds, despite this variation, the underlying trends are also meaningful. “There are themes of life that are deep-seated and fundamental.”

All the business of life needs energy. So if you know the rate at which an organism burns fuel—or if you know how big and hot it is, and apply the metabolic theory—you can make a suite of predictions about its biology, such as how fast it will grow and reproduce, and how long it will live.

**Figure 3:**
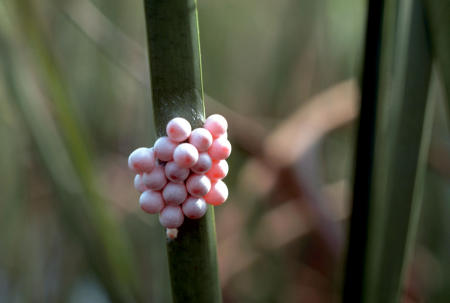
Apple Snail Eggs The hatching times for egg-laying animals, including birds, fish, amphibians, insects, and plankton—or even these Apple Snail eggs—turn out to follow the same relationship. (Photo: Gary M. Stolz, U. S. Fish and Wildlife Service)

By correcting for mass and temperature, Brown, Gillooly, and their colleagues believe they have revealed underlying similarities in all the rates of life. The hatching times for egg-laying animals, including birds, fish, amphibians, insects, and plankton, turn out to follow the same relationship—if a fish egg were the same size and temperature as a bird egg, it would take equally long to hatch (Figure 3). The same goes for growth: a tree and a mammal of equal size and temperature would gain mass at the same speed. And size and temperature even explain much of the variation in mortality rates between species—which one might have thought to be strongly dependent on external factors such as predators—perhaps through metabolism's influence on aging processes, such as free-radical damage to the genome.

## One Rule for All?

If all organisms work in the same way, understanding individual biology offers an obvious route to explaining nature's patterns—ecological processes become a kind of meta-metabolism. Indeed, the team has used their theory to predict the flux of carbon dioxide through forests—a measure more usually used to determine individual metabolic rate. They have also found that body size and temperature predict the densities and growth rates of populations. So hotter environments should support lower population densities, as each individual consumes resources more quickly, leaving less to go round. One thing that does not scale with a quarter power of body size is the area of animals' home ranges; this increases more or less linearly with body size. But in October, Brown, along with researchers from Princeton University and the Institute of Zoology in London, published a model that brought this, too, into the metabolic theory. They borrowed another trick from physics, using an equation that describes colliding gas molecules to model the interactions between neighboring animals.

Temperature could also explain why biodiversity peaks at the equator, the team believes. Organisms with faster metabolisms have faster mutation rates. So the genomes of smaller, hotter animals change more quickly, and they will also get through their generations more rapidly. One would therefore expect to see more new species created in small organisms and warm environments. The large-scale trend in all these rates—hatching time, individual and population growth, ecosystem metabolism, DNA substitution—is closely proportional to a quarter power of body mass.

In the future, Brown's group plans to examine the dynamics of colonial organisms and societies through the lens of metabolic ecology; instead of capillaries, the terminal units of the networks would become ants, or people. There are also many applied problems within the theory's scope, including some of the most significant human impacts on the biosphere. Carbon emissions and the consequent global warming are increasing both the temperature and nutrient supply. And exploited populations, such as fisheries often show a decrease in individual size, as larger animals are preferentially killed. Both these would tend to speed up biological processes. Another team of United States and Italian researchers has found that the same model that describes the growth of individuals can also predict the growth of tumors, hinting that metabolic ecology may have medical applications.

Brown hopes that metabolic ecology will one day become an uncontroversial part of researchers' toolkits, like the theories population geneticists use to predict changes in the frequencies of genes. Before that happens, both the theory's proponents and its opponents have years of work ahead of them. Adopting the theory may also require a shift in ecologists' worldview. Most ecologists work by carrying out experimental manipulations on small groups of similar organisms: the warblers in a woodland, for example, or the grasses of a meadow. When they build models, they do so from empirical data, not from physical first principles. The philosophy behind metabolic ecology disconcerts many researchers, says Robinson. “A lot of traditional biologists are uncomfortable with thinking about data in these terms.”

Kozlowski doubts that simple theories can make precise predictions about the behavior of biological systems on large scales. He believes that metabolic ecology risks leading the discipline up a blind alley: “If I'm right, and the basic model contains an error, correcting the results will be a very long process. If they're not right, they'll have done a disservice to ecology.”

But many ecologists are more optimistic that some unifying principles of nature can be found, and that metabolic ecology, and the debate around it, is a step in the right direction. Some think the theory may be part of an even grander idea. Stephen Hubbell, of the University of Georgia, is one of the architects of another idea causing a stir among ecologists. Called neutral ecology, it proposes a general explanation of how competition between individuals produces the dynamics of birth, death, and migration seen in ecosystems, and its predictions match closely the abundance and diversity of species in the wild. He believes that metabolic and neutral ecology can become elements of some larger theoretical framework.

“I've never been more excited in my life,” says Hubbell. “Ecology now is like quantum mechanics in the 1930s—we're on the cusp of some major rearrangements and syntheses. I'm having a lot of fun.”
